# Simultaneous and sensitive determination of ascorbic acid, dopamine and uric acid via an electrochemical sensor based on PVP-graphene composite

**DOI:** 10.1186/s12951-020-00672-9

**Published:** 2020-08-10

**Authors:** Yiyong Wu, Peihong Deng, Yaling Tian, Jinxia Feng, Jingyun Xiao, Junhua Li, Jun Liu, Guangli Li, Quanguo He

**Affiliations:** 1 School of Life Science and Chemistry, Hunan University of Technology, Zhuzhou, 412007 China; 2 Key Laboratory of Functional Metal–Organic Compounds of Hunan Province; Key Laboratory of Functional Organometallic Materials of Hunan Provincial Universities, Department of Chemistry and Material Science, Hengyang Normal University, Hengyang, 421008 China; 3 School of Materials Science and Energy Engineering, Foshan University, Foshan, 528000 China

**Keywords:** Simultaneous determination, Polyvinylpyrrolidone, Ascorbic acid, Dopamine, Uric acid, Electrochemical sensor

## Abstract

A method with high sensitivity, good accuracy and fast response is of ever increasing importance for the simultaneous detection of AA, DA and UA. In this paper, a simple and sensitive electrochemical sensor, which based on the polyvinylpyrrolidone (PVP)-graphene composite film modified glassy carbon electrode (PVP-GR/GCE), was presented for detecting ascorbic acid (AA), dopamine (DA) and uric acid (UA) simultaneously. The PVP-GR/GCE has excellent electrocatalytic activity for the oxidation of AA, DA and UA. The second-order derivative linear sweep voltammetry was used for the electrochemical measurements. The peak potential differences of DA-AA, DA-UA, and UA-AA (measured on the PVP-GR/GCE) were 212, 130 and 342 mV respectively. Besides, the over potential of AA, DA and UA reduced obviously, so did the peak current increase. Under the optimum conditions, the linear ranges of AA, DA and UA were 4.0 μM–1.0 mM, 0.02–100 μM, and 0.04–100 μM, respectively. The detection limits were 0.8 μM, 0.002 μM and 0.02 μM for AA, DA, and UA. The electrochemical sensor presented the advantages of high sensitivity and selectivity, excellent reproducibility and long-term stability. Furthermore, the sensor was successfully applied to the analysis of real samples.
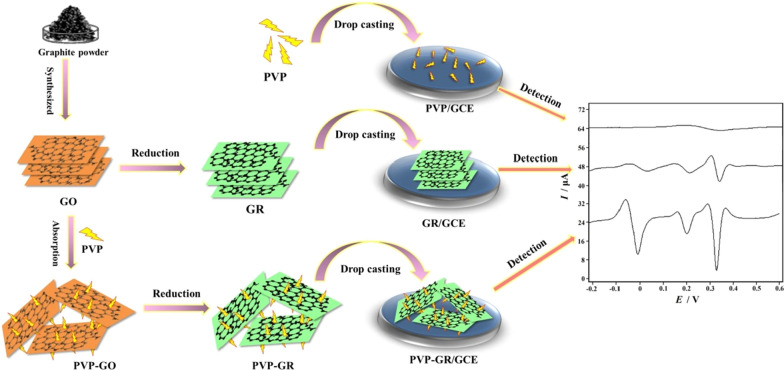

## Introduction

As the key small biomolecules in the physiological process of human metabolism, ascorbic acid (AA), dopamine (DA) and uric acid (UA) generally coexist in biological matrix at the same time. Abnormal levels of AA, DA and UA will lead to some disorders and diseases, such as mental illness, Parkinson’s disease, hyperuricemia and leukemia [1–4]. Therefore, it is of great significance to establish a sensitive and rapid method for the simultaneous detection of AA, DA and UA for the study of their physiological signal procession and diagnostic applications. However, since the signals of AA, DA and UA are overlap, it is difficult to obtain the content of these three compounds separately in the extracellular fluid of the central nervous system, and serum where they usually coexist.

For the determination of AA, DA and UA, several methods have been developed, such as using high performance liquid chromatography [5], chemiluminescence [6], capillary electrophoresis [7] and ultraviolet visible spectroscopy [8]. In recent years, electrochemical methods have been widely concerned because of their rapid response, high sensitivity, good stability, excellent selectivity and so on [9]. However, it’s not able to determine the AA, DA and UA separately on bare electrode without any modifications since the oxidation potentials of these three compounds are approximate [10]. Recently, various materials have been used in electrode modification for overcoming this problem [11–13].

In recent years, various nanomaterials have been well applied in different fields [14]. Among them, graphene (GR) is a two-dimensional carbon nanomaterial composed of carbon atoms in a hexagonal lattice. It is a single-layer film of carbon atoms in hexagonal lattice. With the strengths of excellent conductivity, large specific surface area, wide potential window and low cost, it is considered as a potential nanomaterial for the design of new sensors [15–23]. However, the interaction between individual GR may not only lead to irreversible aggregation, but also even restock to form graphite, so that the dispersibility of GR in water and some other solvents is poor and the application is seriously limited. Thus, it is inadvisable to apply GR directly to simultaneous detection of small biomolecules. To address these issues, GR-based nanocomposites have been exploited in recent years to modify electrodes for simultaneously detecting the AA, DA and UA. For instance, Tukimin et al. were centered on using poly (3,4-ethylenedioxothiophene)/reduced graphene oxide/manganese dioxide composite as electrode material to construct electrochemical sensor for simultaneous detection of AA, DA and UA, with the detection limits as low as 1.00 μM, 0.02 μM, and 0.05 μM [24]. Besides, Tian et al. reported the detection of AA, DA and UA by gold nanoparticles/β-cyclodextrin/graphene nanocomposite modified electrode (detection limits were 10 μM, 0.15 μM and 0.21 μM) [25]. Recently, Niu and his colleagues successfully proposed a sensor based on stacked graphene platelet nanofibers/ionic liquids/chitosan to test AA, DA and UA simultaneously [26]. Moreover, Sun’s team used graphene/Pt nanocomposites as electrode modifiers for simultaneous determination of AA, DA and UA [27], and Lian et al. developed a new sensing platform based on tryptophan-functionalized GR modified electrode for detecting AA, DA and UA simultaneously [28]. In spite of the successful separation from the oxidation potentials of these three species by the above studies, some nanomaterials are relatively expensive, and some synthesis process is complex and time-consuming. Therefore, it is necessary to explore a preferable modified electrode which is easy to fabricated, cost-effective, and sensitive to achieve the simultaneous detection of these three substances.

Polyvinylpyrrolidone (PVP), as a kind of non-toxic polymer and non-ionic surfactant, exhibits ideal features for the electrode modification due to its strong adsorption capacity towards phenolic compounds due to the hydrogen bonding between imide moieties of polymer and hydroxyl group in phenolic compounds [29]. What’s more, it was found that PVP was able to make the carbon atoms less prone to aggregation in GR nanosheets. Thus, the dispersion and stability of PVP-GR composite boosted obviously compared with pure GR. Herein, for the accurate detection of AA, DA and UA simultaneously on the same electrode, a simple detection method using PVP-GR/GCE was proposed. The sensor with good catalytic activity for the oxidation of AA, DA and UA could help to distinguish these three species with similar redox potentials. Furthermore, the electrochemical behaviors of AA, DA and UA at the PVP-GR/GCE were studied in detail.

## Experimental

### Chemicals and solutions

The solutions received from Sinopharm Chemical Reagent Co., Ltd. Graphite, polyvinylpyrrolidone, hydrazine solution (80 wt %), ammonia solution (25 wt %), hydrogen peroxide solution (30 wt %), ascorbic acid (AA), dopamine (DA) and uric acid (UA) were acquired from Aladdin Chemical Reagent Co., Ltd., China. Other reagents were analytical grade and adopted as received. All human urine samples were collected from the laboratory personnel. All aqueous solutions were prepared with ultra-pure water (specific resistance of 18 MΩ cm). 0.1 M phosphate buffered solutions (PBS, pH 6.0) was used throughout this study.

### Instruments and characterizations

Cyclic voltammetry (CV) measurements were performed on a CHI 660E electrochemical workstation which was produced from Chenhua Instrument Co. Ltd. Shanghai, China. A model JP-303E polarographic analyzer from Chengdu Instrument Factory in China was used for quantitative analysis of the samples by the second-order derivative linear sweep voltammetry (SDLSV). The saturated calomel electrode (SCE), platinum wire electrode and modified glassy carbon electrode (d = 4 mm) were used as the reference electrode, auxiliary electrode and working electrode respectively in this electrode system. The pH values were measured with a combined glass electrode on the pH-3c Model pH meter, which was purchased from Leichi Instrument Factory, Shanghai, China. Scanning electron microscope (EVO10, ZEISS, Jena, Germany) images were obtained under 2.0 kV accelerating voltage. A high-speed centrifuge (maximum speed 16000r/min) was provided by Changsha Yingtai Instrument Co., Ltd., China.

### Preparations of GR and PVP-GR composite

First of all, the synthesis of graphite oxide was prepared by natural graphite powder [30, 31]. Secondly, 100 mg of graphite oxide was dissolved in ultra-pure water (100 mL) and exfoliated to be graphene oxide (GO) under sonication for 2 h to obtain a light yellow solution. Subsequently, centrifugation was performed to remove any impurities (usually in trace amount) adhered to graphite oxide. 10.0 mg PVP was added to a 20.0 mL GO solution, then stirred for 10 min at room temperature. Following on that, 20 μL hydrazine hydrate solution and 80 μL ammonia solution were added, and the mixture was reacted at 95° C for 1 h. At the end of the reaction, stable PVP-GR composite was obtained. As a control, the preparation method of GR was the same as above, except that no PVP was added.

### Electrode preparation

**Scheme 1:**
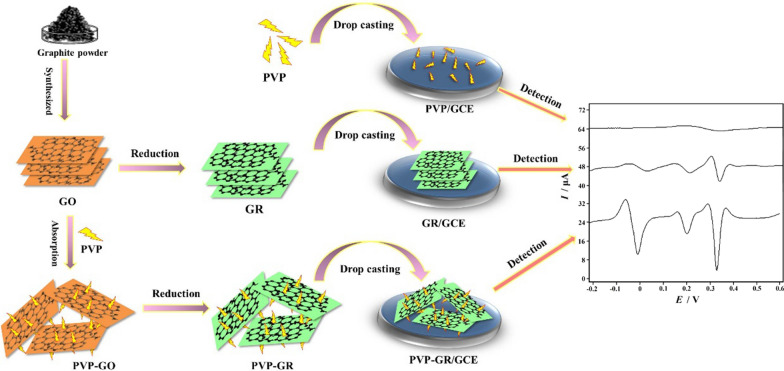
Diagram for the preparation of PVP/GCE, GR/GCE and PVP-GR/GCE and the electrochemical behaviors of AA, DA and UA

Before modification, GCE was polished on a polishing cloth with 0.3 μm and 0.05 μm alumina slurries in sequence, then washed thoroughly with water between each polishing step. Subsequently, the GCE was sonicated with water and ethanol in turn. Finally, the GCE was dried under an infrared lamp. In order to prepare PVP-GR/GCE, PVP-GR dispersion (10 μL) was carefully dropped on the electrode surface and dried under an infrared lamp. Besides, PVP/GCE and GR/GEC were also prepared by 10 μL of 1.0 mg mL^−1^ PVP and 10 μL of 1.0 mg mL^−1^ GR for comparison. The whole electrode preparation process was shown in Scheme 1.

### Electrochemical measurements

Electrochemical experiments were carried out by CV in a 10.0 mL 0.10 M PBS (pH 6.0) containing the single or mixed components of AA, DA and UA. The preconcentration of analytes was performed on the PVP-GR/GCE with an accumulation potential (0 V) under constant stirring. After a certain period of time, CV or SDLSV was performed in quiescent solution. For sample analysis, SDLSV was run with a positive going potential scan (−0.2–0.60 V) under optimal conditions. All the electrochemical measurements were carried out at room temperature without removing oxygen from the solution.

## Results and discussion

### SEM Characterizations of GR and PVP-GR

**Fig. 1:**
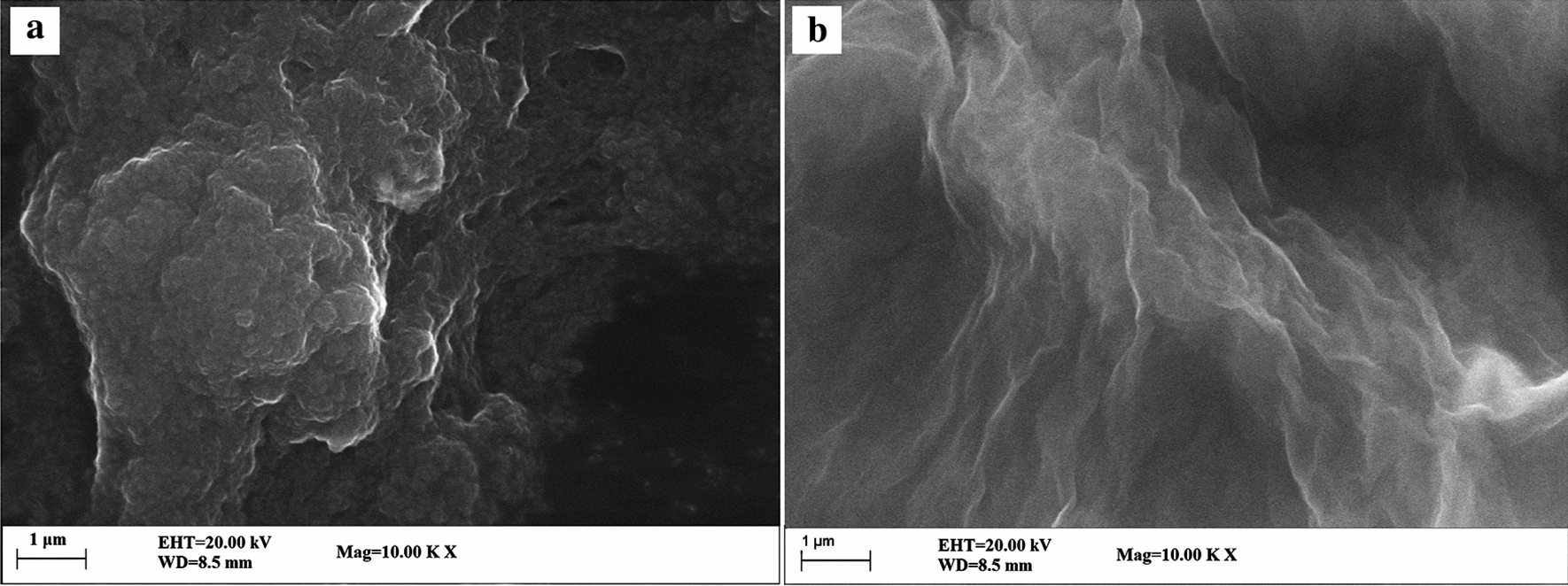
SEM images of GR (**a**) and PVP-GR (**b**). Accelerating voltage: 20 kV

The surface morphology of GCE modified with GR and PVP-GR composite was investigated by SEM. It can be clearly observed the typically crumpled and wrinkled structure of GR in Fig. 1a. Figure 1b shows the SEM image of PVP-GR composite. The wrinkles on the GR sheets are clearly observed. This wrinkled nature of GR is highly beneficial in maintaining a high surface area on the electrode since the sheets cannot readily collapse back to a graphitic structure [32]. Most importantly, it could indicate that the dispersity of PVP-GR composite was much better, which was extremely essential for nanomaterials dispersed in solution and for their further application [33]. Therefore, it is greatly expected that the performance of the sensor is improved by the PVP-GR composite.

**Fig. 2:**
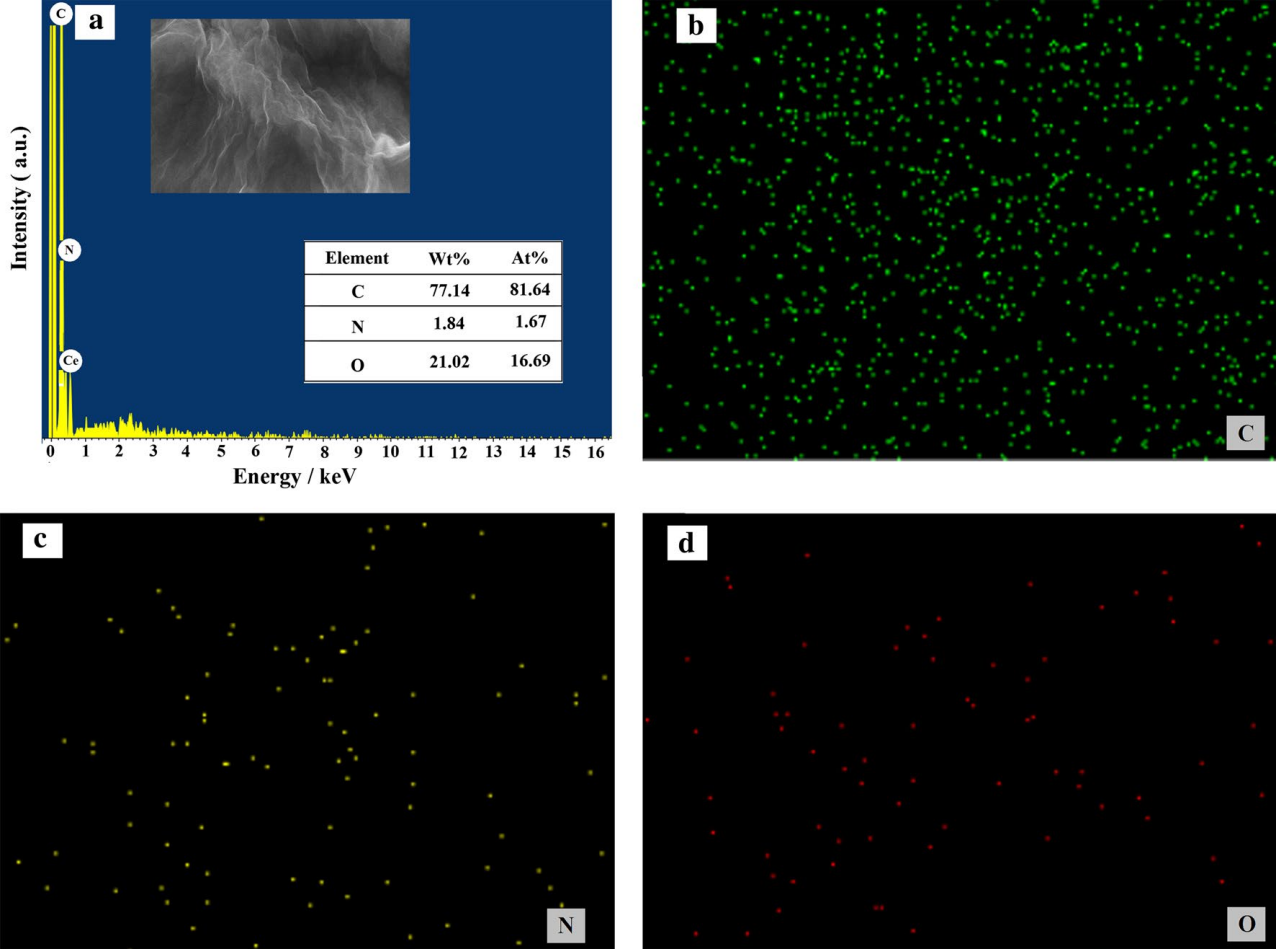
EDS spectrum (**a**) and typical SEM image (insert of A) of PVP- GR nanocomposite together with the corresponding element mapping images of C (**b**), N (**c**) and O (**d**)

The element composition and distribution of PVP-GR nanocomposite were analyzed by EDS. As shown in Fig. 2, a series of peaks related to the species of C, O, and N were observed in the survey spectrum, which confirmed the presence of these chemical elements in the product. At the same time, the element mapping images (Fig. 2b–d) corresponding to the insert of Fig. 2a indicated that, C, O and N were uniformly dispersed on the nanocomposite, and C, O and N were present in the structure at 77.14, 21.02 and 1.84 wt % and 81.64, 16.69 and 1.67 At %, respectively.

### Electrochemical characterization by CV

**Fig. 3:**
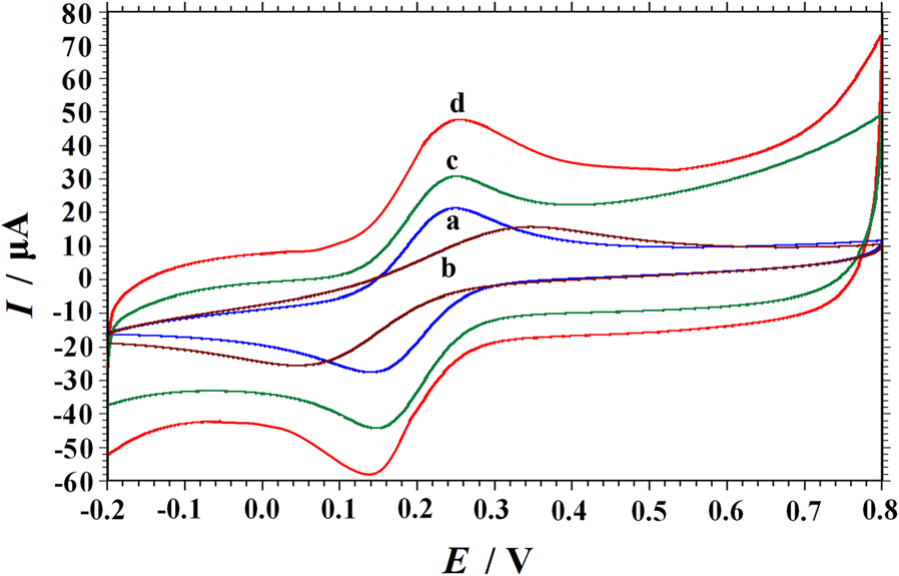
CVs of different electrodes: **a** GCE, **b** PVP/GCE, **c** GR/GCE and **d** PVP-GR/GCE. Solution: 1.0 mM K_3_[Fe(CN)_6_] in 1.0 M KCl; Scan rate: 0.1 V s^−1^

The electrochemical method was used to characterize the charge transfer characteristics of PVP-GR/GCE. The cyclic voltammograms (CVs) showed in Fig. 3 was obtained in 1.0 mM K_3_[Fe(CN)_6_] solution containing 1.0 M KCl at bare GCE, PVP/GCE, GR/GCE and PVP-GR/GCE. Although the appearance of a pair of redox peaks showed on the electrodes, their potential differences (Δ*E*p) were totally different. The potential difference of bare GCE (curve a), PVP/GCE (curve b), GR/GCE (curve c) and PVP-GR/GCE (curve d) was 109 mV, 298 mV, 103 mV and 105 mV, respectively. Apparently, a pair of well-defined quasi-reversible redox peaks appeared at the PVP-GR/GCE, and the peak current signal of PVP-GR/GCE was observably larger than the other three. According to Randles–Sevcik formula (1) [34]:

1 $$I_{p} \, = \,2.69\, \times \,10^{5} n^{3/2} \,A_{eff} D^{1/2} \nu^{1/2} C$$

where *I*_p_ is the peak current (A), *n* is the number of electrons transferred, *A*_eff_ is the effective area (cm^2^), *D* is the diffusion coefficient of K_3_Fe(CN)_6_ (7.6 × 10^−6^ cm^2^ s^−1^) [35], *v* is the scan rate (V s^−1^) and *C* corresponds to the bulk concentration of K_3_Fe(CN)_6_ (mol cm^−3^). According to the above formula, the electroactive surface area of PVP-GR/GCE is calculated as 0.1996 cm^2^, which is larger than that of GR/GCE (0.1493 cm^2^). The results released that the PVP-GR composite promoted the electron transfer rate, which may be contribute to the excellent conductivity and large specific surface area of PVP-GR modified on GCE surface.

### Electrochemical behaviors of AA, DA and UA at PVP-GR/GCE

**Fig. 4:**
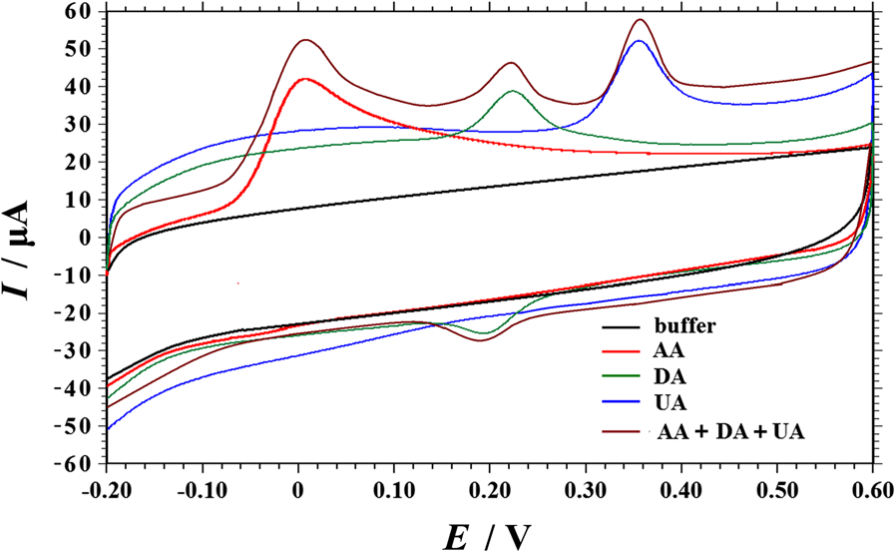
CVs obtained on the PVP-GR/GCE in 0.1 M PBS (pH 6.0) in the absence (black) and presence of 1.0 mM AA (red), 20 μM DA (green), 50 μM UA (blue) and a mixture solution containing 1.0 mM AA, 20 μM DA and 50 μM UA (brown), respectively. Scan rate: 100 mV s^−1^

Figure 4 depicted the CV response in 0.1 M PBS (pH 6.0) obtained at the GR-PVP/GCE in the potential range of 0.0 to 1.2 V. As can be seen, no redox peaks were observed at the PVP-GR/GCE in the blank solution, indicating that the PVP-GR/GCE is non-electroactive in the selected potential region and there is no distribution of other reducing substances in the tested solution. The electrochemical behaviors of AA, DA, and UA at the PVP-GR/GCE were studied by CV in 0.1 M PBS (pH 6.0) containing AA, DA, UA and a mixture of AA, DA and UA respectively. In order to make the three substances have an obvious current response in the same range when determined simultaneously, 1.0 mM AA, 20 μM DA, and 50 μM UA were selected in our work. Figure 4 showed the irreversible electro-oxidation of AA and UA, while a reversible response of DA was exhibited at the PVP-GR/GCE. The ${\Delta}E_{p} \, = \left| {E_{pa} \, - \,E_{pc} } \right|\,$ is 29 mV and *I*_pa_/*I*_pc_ = 0.89 for DA at 100 mV s^−1^. It can be detected in Fig. 4 that the anodic peaks of AA, DA and UA appeared approximately at the potential of 8, 194, and 356 mV, respectively. These data showed that it is feasible to discriminate these three species on the PVP-GR/GCE.

**Fig. 5:**
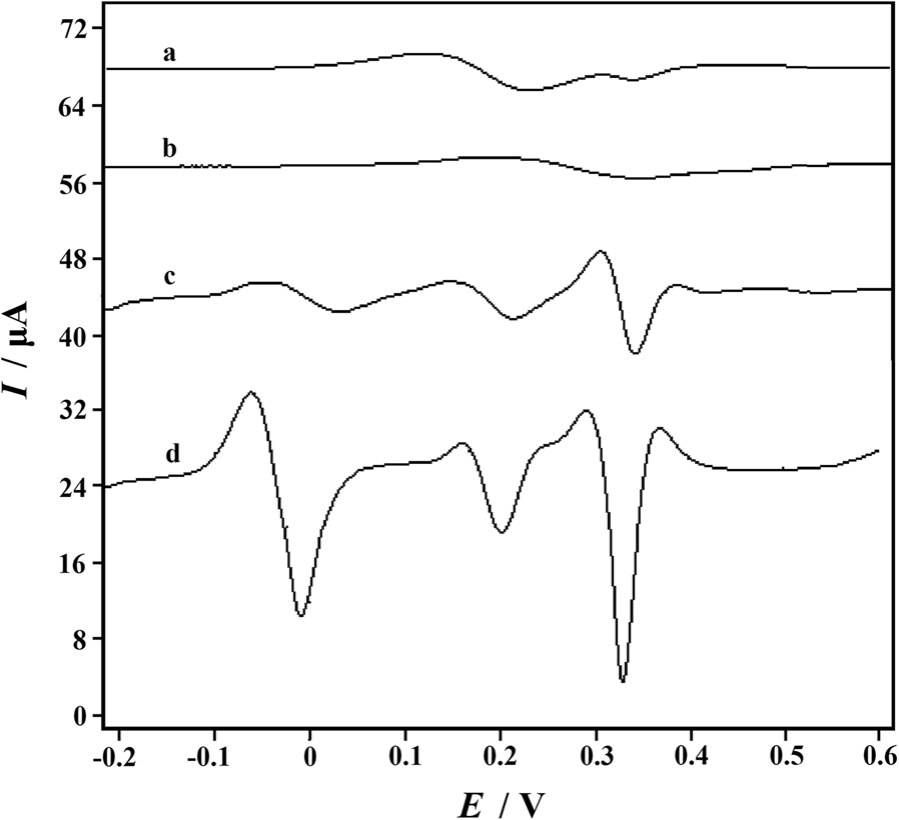
Second-order derivative linear scan voltammograms (SDLSVs) obtained at GCE (**a**), PVP/GCE (**b**), GR/GCE (**c**) and PVP-GR/GCE (**d**) in 0.1 M phosphate buffer solution (pH 6.0) containing 1.0 mM AA, 20 μM DA and 50 μM UA. (0.0 V for the accumulation potential; 60 s for the accumulation time; 0.1 V s^−1^ for the scan rate)

In order to demonstrate that the PVP-GR/GCE could simultaneously detect these three compounds, the SDLSV curves of the phosphate buffer solution (pH 6.0) with the simultaneous presence of 1.0 mM AA, 20 μM DA and 50 μM UA were also recorded. Figure 5 displayed the corresponding voltammetric curves of the mixture obtained at bare GCE (curve a), PVP/GCE (curve b), GR/GCE (curve c) and PVP-GR/GCE (curve d) respectively. On the bare GCE, there are two peaks at 232 mV and 340 mV, corresponding to the overlapping voltammetric response of AA and DA and the oxidation of UA. The peak current is extremely weak (*I*_p_ = 3.900 μA and 0.5125 μA), which indicates that it is not feasible to detect these three small biomolecules simultaneously on bare GCE. At the PVP/GCE. It can be only observed a weak and broad peak at 0.35 V (*I*_p_ = 1.938 μA), which suggests that the oxidation peaks of that three may be not separated well. This is mainly due to the poor conductivity of PVP, which leads to the retardance of electron transfer. However, the oxidation peaks of AA, DA and UA presented at 34 mV (*I*_p_ = 3.138μA), 214 mV (*I*_p_ = 5.263μA), and 344 mV (*I*_p_ = 9.325μA) on the GR/GCE, make it possible to detect these species simultaneously since GR has good catalytic ability for that three species. Three oxidation peak potentials of −10 mV (AA), 202 mV (DA) and 332 mV (UA) were obtained on the GR-PVP/GCE, which shifted negatively compared with GR/GCE. Distinctly, for GR-PVP/GCE, the peak currents increased significantly (*I*_p_ = 20.21μA, 10.50 μA and 28.17μA). On one hand, with the good electrocatalytic activity and conductivity, GR could accelerate the electron transfer rate of AA, DA and UA on the electrode surface. On the other hand, it is well known that most unique properties are only associated with individual form. Therefore, preventing aggregation is particularly important for GR. The addition of PVP can effectively prevent the aggregation of GR, so that the PVP-GR composite can be well dispersed in water. When it was modified on the GCE surface, it could effectively increase the GCE specific surface area. Therefore, the synergistic effect of PVP and GR significantly increased the peak signals of AA, DA and UA, and improved the sensitivity of the determination.

### The effect of solution pH

**Fig. 6:**
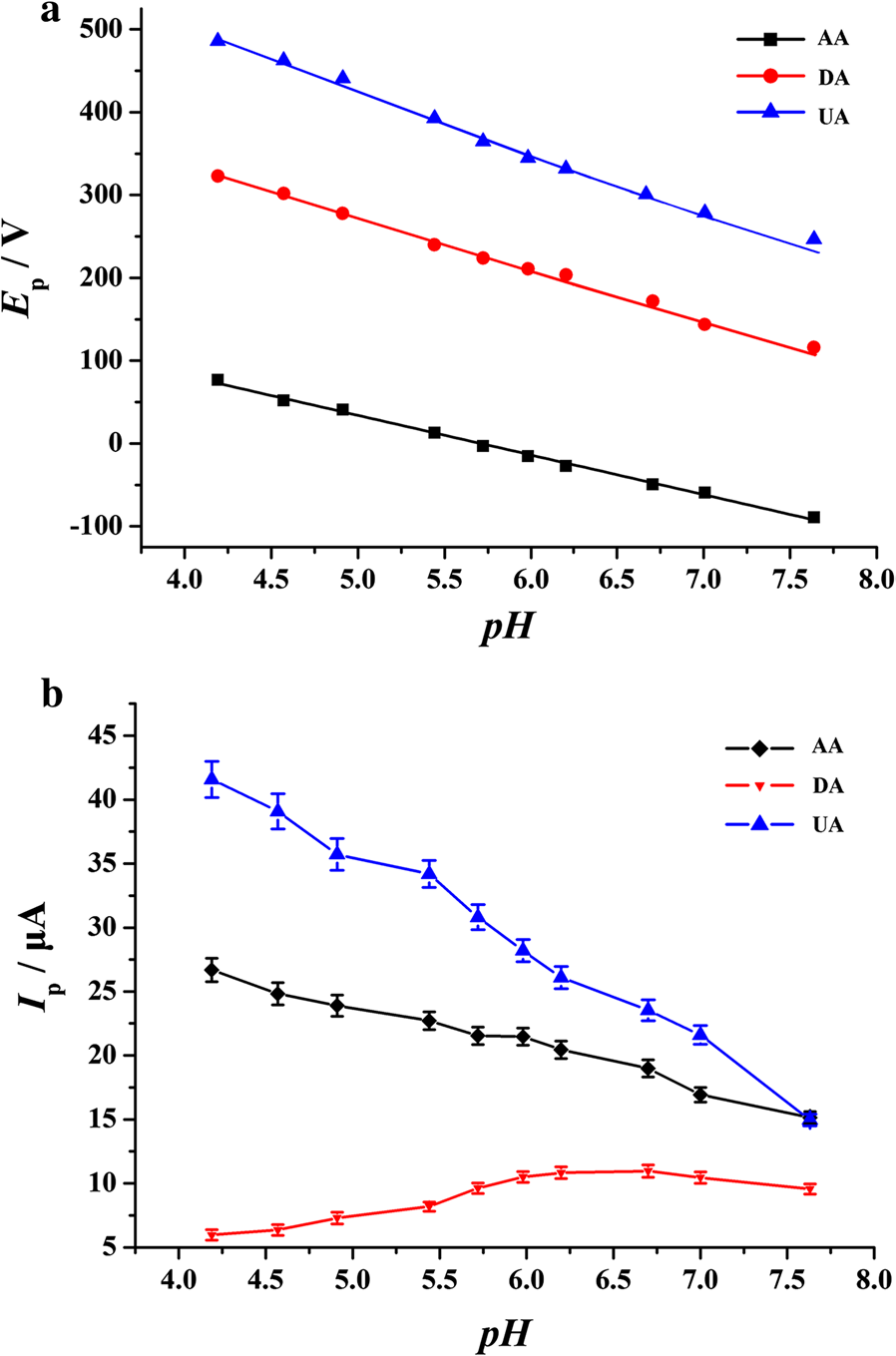
Influences of pH value on the oxidation peak potential (**a**) and oxidation peak current (**b**) of 1.0 mM AA, 20 μM DA and 50 μM UA obtained at PVP-GR/GCE, respectively. The scan rate was 100 mV s^−1^. The concentration of PBS was 0.1 M (pH 6.0). Error bars represent SD, n = 3

It is well-know that the pH value of the analytical solution is of great significance to an experimental exploration, since the protons are involved in the electrode reactions. Therefore, the effects of pH value on the oxidation potential and current responses at PVP-GR/GCE were investigated in 1.0 mM AA, 20 µM DA and 50 µM UA, respectively. The acidity of the solution could affect the oxidation peak potential of AA, DA and UA. The result showed in Fig. 6a. With increasing the pH value, the oxidation potential of AA, DA and UA shifted negatively. For AA, the linear regression equation was *E*_pa_ (mV) = 0.2726−0.04792 *pH* (R^2^ = 0.9970), while that one of DA was *E*_pa_ (mV) = 0.5760−0.06095 *pH* (R^2^ = 0.9961) and UA was *E*_pa_ (mV) = 0.7607−0.06785 *pH* (R^2^ = 0.9829). These results illustrated that electrochemical oxidation of AA, DA and UA followed Nerst equation, and the electrochemical reactions of them are all two-proton and two-electron transfer processes [36, 37]. At the same time, as shown in Fig. 6b, with the increase of solution pH value, the peak currents of AA and UA gradually reduced. However, on the contrary, the peak current of DA increased with the pH value reached to 6.0. However, the current of DA tended to decrease when the pH value was over 6.7. Thus, the larger peak current appeared at the pH value of 6.0–6.7. In order to determine these three substances sensitively at the same time, and considering the physiological pH value of 7.0, the pH value of 6.0 was selected for further study [38].

### The effect of scan rate

**Fig. 7:**
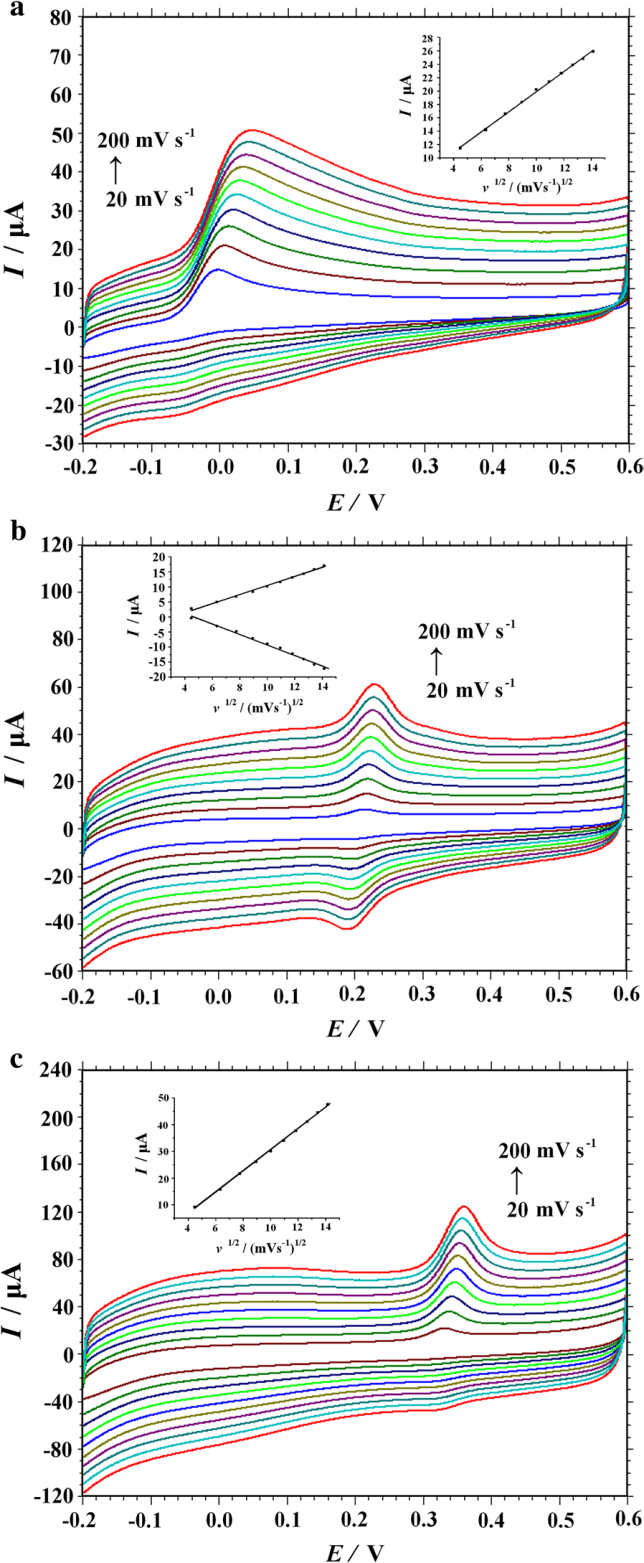
CVs obtained on PVP-GR/GCE in phosphate buffer solution PBS (0.1 M, pH 6.0) in the presences of 1.0 mM AA (**a**), 20 μM DA (**b**) and 50 μM UA (**c**) respectively by different scan rates (20, 40, 60, 80, 100, 120, 140, 160, 180, 200 mV s^−1^)

Scan rate is one of the factors to study the electrochemical behavior. In this work, the electrochemical behavior of AA, DA and UA was further explored on the PVP-GR/GCE. Figure 7 showed the relationship between the scan rates and the currents of AA, DA and UA, respectively. In the range of 20–200 mV s^−1^, the peak currents of the three compounds increased linearly with the scan rate. For AA (Fig. 7a) and UA (Fig. 7c), it is clearly observed that only one anodic peak presented in the voltammogram and the relationship between the peak current and the square root of scanning rate (Fig. 7a, c, inset). The linear regression equation of AA was *I*pa (µA) = 4.9231 + 1.4961*v*^1/2^ ((mV s^−1^)^1/2^) (R^2^ = −0.9988), and that one of UA was *I*pa (µA) = −9.1577 + 3.9751*v*^1/2^ ((mV s^−1^)^1/2^) with a high correlation coefficient of R^2^ = −0.9992. The results indicated that the diffusion controlled behavior dominated the electrode process of AA and UA. However, for DA (Fig. 7b), there were an anodic peak and a cathodic peak appeared in the CVs. Besides, the anodic and cathodic peak current of DA are linearly related to the scan rate (Fig. 7b, inset). The corresponding linear regression equations were *I*pa (µA) = 1.919 + 0.0782*v*(mVs^−1^) (R^2^ = 0.9948) and *I*pc (µA) = 0.756−0.0921*v* (mV s^−1^) (R^2^ = 0.9955), suggesting that the electrochemical oxidation of DA at PVP-GR/GCE was an absorption-controlled process. The fact might be attributed to the effective π-π conjugation between the aromatic moieties of DA and GR [39, 40].

### Investigation of accumulation potential and accumulation time

**Fig. 8:**
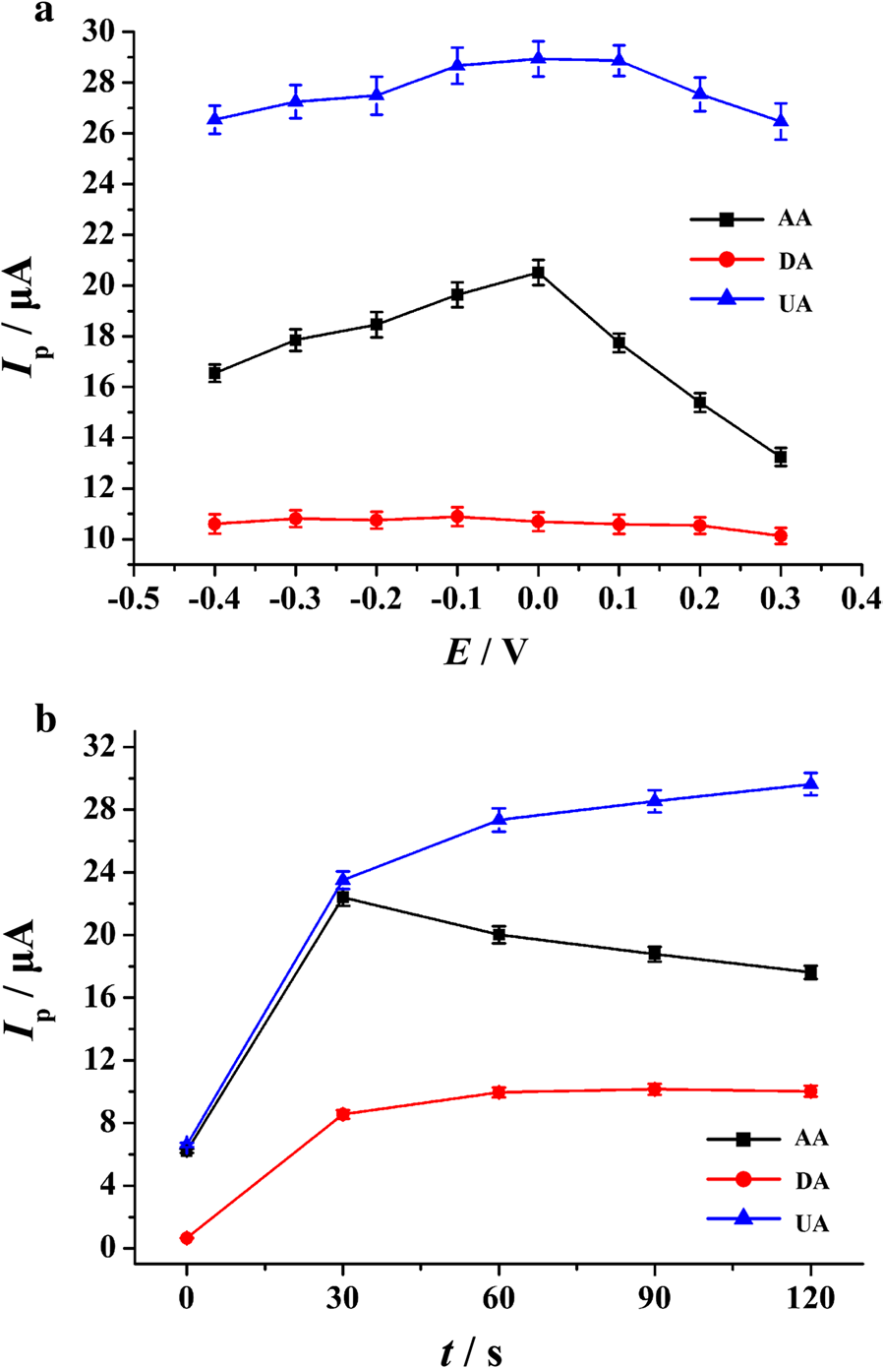
The influence of accumulation potential (**a**) and time (**b**) on the oxidation currents of 1.0 mM AA, 20 μM DA and 50 μM UA. Error bars represent SD, n = 3

Accumulation potential and accumulation time are also the important conditions for this work. The influence of accumulation potential and accumulation time on the peak currents of AA, DA and UA was studied by SDLSV. As the Fig. 8a presented, the peak currents of AA and UA decreased slightly after increasing gradually as the potentials shifted from −0.40 to 0.30 V. However, the change of the peak current of DA was a little different in comparison, it was not affected by the accumulation potential. Therefore, according to the observation of the Fig. 8a, the accumulation potential of 0.0 V was considered to be beneficial to obtain the maximum peak currents for simultaneous detection of AA, DA and UA. In addition, time also made a difference on pre-concentration. According to Fig. 8b, it could be described that the peak currents of AA, DA and UA increased rapidly with the increase of accumulation time. The maximum peak currents of AA, DA and UA were obtained at 30 s, 60 s and 90 s, respectively. For obtaining a stable peak with higher sensitivity and shorter analysis time, it was suggested that setting 60 s as the accumulation time to detect AA, DA, and UA is optimum.

### Simultaneous determination of DA, AA and UA

**Fig. 9:**
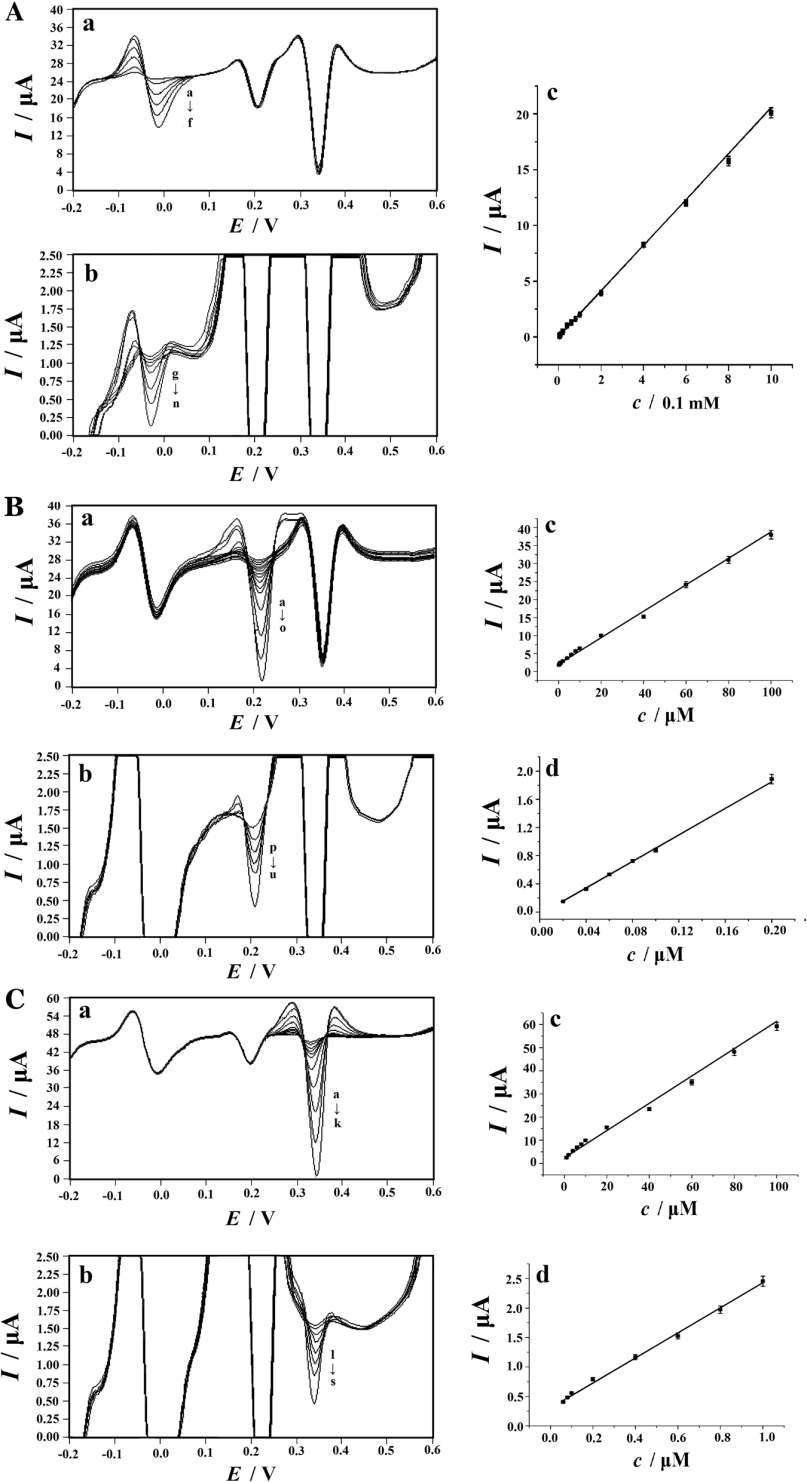
**A** SDLSVs of AA at the PVP-GR/GCE in the presence of 20 μM DA and 50 μM UA. a AA concentrations (from a to f): 100, 200, 400, 600, 80, 1000 μM; b AA concentrations (from g to n): 4, 6, 8, 10, 20, 40, 60, 80 μM. c Plots of peak height vs. AA concentration in the range of 4–1000 μM. **B** SDLSVs of DA at the PVP-GR/GCE in the presence of 1.0 mM AA and 50 μM UA. a DA concentrations (from a to o): 0.2, 0.4, 0.6, 0.8, 1.0, 2.0, 4.0, 6.0, 8.0, 10, 20, 40, 60, 80 and 100 μM; b DA concentrations (from p to u): 0.02, 0.04, 0.06, 0.08, 0.1, 0.2 μM; c Plots of peak height vs. DA concentration in the range of 0.2–100 μM and d in the range of 0.02–0.2 μM. **C** SDLSVs of UA at the PVP-GR/GCE in the presence of 1.0 mM AA and 20 μM DA. a UA concentrations (from a to k): 1.0, 2.0, 4.0, 6.0, 8.0, 10, 20, 40, 60, 80, and 100 μM; b UA concentrations (from l to s): 0.04, 0.06, 0.08, 0.1, 0.2, 0.4, 0.6, 0.8 μM; c Plots of peak height vs. UA concentration in the range of 1.0–100 μM and d in the range of 0.04–1.0 μM. 0.1 M PBS (pH 6.0) for the supporting electrolyte, 0.0 V for the accumulation potential, 60 s for the accumulation time, 0.1 V s^−1^ for the scan rate. Error bars represent SD, n = 3

Compared with CV, SDLSV has higher current sensitivity and better resolution, it was a good electrochemical technique for the simultaneous detection of AA, DA and UA. Under the optimized conditions, the potential range of −0.2 V to 0.6 V was selected for the measurements. When the concentration of one substance changed, the concentrations of the other two substances remained unchanged. It can be obviously described that the current response of the target molecules (AA, DA, or UA) enhanced linearly with the increasing of the corresponding target molecules concentration. According to Fig. 9, when the concentration range of AA was 4.0 μM −1.0 mM with the presence of 20 μM DA and 50 μM UA, the electrochemical response of AA grew linearly as the concentration of AA increased (Fig. 9A). *I*_pa_ (μA) = 0.00726 + 0.02054*c* (μM) (R = 0.9891) was the regression equation of AA. Besides, as shown in Fig. 9B, the oxidation peak currents of DA were obtained in various concentration of DA (0.02–0.2 μM and 0.2–100 μM), with the presence of 1.0 mM AA and 50 μM UA. A positive correlation between the increase in DA concentration and the increase of current response of DA was found. The regression equations were *I*_pa_ (μA) = 0.040 + 9.4239 *c* (μM) (R = 0.9985) and *I*_pa_ (μA) = 2.0587 +0.3685*c* (μM) (R = 0.9828). Similarly, it was revealed in Fig. 9C that the signal of UA increased linearly with increasing the concentration of UA from 0.04–1.0 μM and 1.0–100 μM, while the concentrations of AA and DA was 1.0 mM and 20 μM in the analytical solutions. The regression equation were *I*_pa_ (μA) = 0.3083 + 2.1242*c* (μM) with a high correlation coefficient of R = 0.9901, and *I*_pa_ (μA) = 2.3954 + 0.5897*c* (μM) (R = 0.9669) with the detection limit of 0.02 μM. The limit of detection (LOD) was calculated by using IUPAC (International Union of Pure and Applied Chemistry) definitions, using the following equation:

$$LOD\, = \,3s/m.$$

where *s* is the standard deviation of the blank signal, and *m* was the slope of the calibration straight line in the low concentration range. The SD of blank sample for AA, DA and AA were 0.0005460, 0.0006434 and 0.01315, respectively. The slopes of the calibration straight line in the low concentration range for AA, DA, UA were 0.02054 μA/μM, 9.4239 μA/μM, 2.1242 μA/μM, respectively. Therefore, the LOD of AA, DA and UA was calculated to be 0.8 μM, 0.002 μM and 0.02 μM, respectively.

The results by this method were compared with those in the literature (Table 1). By contrast, it can be easily found that lower detection limit, higher sensitivity and wider linear range were obtained at PVP-GR/GCE.

**Table 1:** Comparison of analytical performance of PVP-GR/GCE with other modified electrodes in the literature

Modified electrode	Technique	Linear range/μM			Detection limit/μM			Reference
		AA	DA	UA	AA	DA	UA	
Pd/CNF-CPE^a^	DPV^i^	50–4000	0.5–160	2.0–200	15.0	0.20	0.70	[11]
PGE-DA^b^	DPV	25–500	1.0–20	2.5–30	13.0	0.11	1.40	[12]
MCNT/CCE^c^	DPV	15–800	0.5–100	0.55–90	7.71	0.31	0.42	[13]
PrGO/MnO_2_/CCE^d^	DPV	1–800	0.03–45	0.3–80	1.00	0.02	0.05	[20]
AuNPs-β-CD–GR/GCE^e^	SWV^j^	30–2000	0.50–150	0.5–60	10	0.15	0.21	[21]
SGNF/IL/CS/GCE^f^	DPV	30–350	0.05–240	0.12–260	14.8	0.04	0.10	[22]
GR-Pt/GCE^g^	Amperometry	0.15–34.4	0.03–8.13	0.05–11.85	0.15	0.03	0.05	[23]
Trp-GR/GCE^h^	DPV	200–12900	0.5–110	10–1000	10.09	0.29	1.24	[24]
PVP-GR/GCE	SDLSV	4.0–1000	0.02–0.2; 0.2–100	0.04–1.0; 1.0–100	0.8	0.002	0.02	This work

^a^Palladium nanoparticle-loaded carbon nanofibers modified carbon paste electrode

^b^Pyrolytic graphite electrode modified into dopamine solution

^c^MWCNT modified carbon-ceramic electrode

^d^Poly(3,4-ethylenedioxythiophene)/reduced graphene oxide/manganese dioxide modified glassy carbon electrode

^e^Gold nanoparticles-β-cyclodextrin-graphene-modified electrode

^f^Stacked graphene platelet nanofibers/ionic liquids/chitosan modified glassy carbon electrode

^g^Graphene/size-selected Pt nanocomposites modified glassy carbon electrode

^h^Tryptophan functionalized graphene modified glassy carbon electrode

^i^Differential pulse voltammetry

^j^Square wave voltammetry

### Reproducibility, repeatability and stability of the modified electrode

Reproducibility, repeatability and stability all played important roles in evaluating the accuracy of the proposed method. Ten equally prepared electrodes were tested in the mixture solution of 1.0 mM AA, 20 μM DA and 50 μM UA, to evaluate the reproducibility of the PVP-GR/GCE by SDLSV. The relative standard deviations (RSD) of the peak currents of AA, DA and UA were 4.8%, 5.7% and 4.2% respectively. Moreover, the repeatability was obtained in the above mixture solution at a single PVP-GR/GCE for seven successive measurements. The RSD values of AA, DA and UA were 2.3%, 3.4% and 2.8%. These results indicated that the reproducibility and repeatability of PVP-GR/GCE was acceptable. When the PVP-GR/GCE was not in use, it was stored in air at 4 ◦C. The stability of the PVP-GR/GCE was studied in 7 days and 15 days respectively. There was no significant change after 7 days storage, and the current maintained 95.4% of the initial current. After 15 days of storage, the current also remained 83.6% compared with the initial current. The results showed that the stability of PVP-GR/GCE was decent.

### Analytical applications

For the evolution of the applicability of the proposed method, AA, DA and UA in human urine samples were analyzed at the PVP-GR/GCE. The samples were diluted 100 times with phosphate buffer solution, and then 1 mL of the diluted urine samples were transferred to a voltammetric cell, and analyzed according to the above-described procedure. The test results were shown in Table 2. No signals of AA and DA were observed while the UA concentrations were 1.58 ~ 3.16 μM. Considered the dilution of the samples, the UA contents in the urine samples were calculated to be 1.58 ~ 3.16 mM. Standard AA, DA and UA solutions were added to the sample solution for recovery test. The results showed that the recoveries of AA, DA and UA were 97.3%–102.5%, 101.8%–105.4% and 97.7%–103.0% respectively. Acceptably and satisfyingly, these results indicated this developed method were validated and reliable. Additionally, the results also showed that AA, DA and UA can be determined by this method individually or simultaneously without interference.

**Table 2:** Determination of AA DA, and UA in human urine samples (n = 4)

Samples^a^	Detected ^a^/µM			Added/µM			Found^b^/µM			Recovery/ %		
	AA	DA	UA	AA	DA	UA	AA	DA	UA	AA	DA	UA
Urine 1	–	–	2.57 (± 0.09)	10.0	10.0	2.0	10.25 (± 0.42)	10.18 (± 0.37)	4.63 (± 0.19)	102.5	101.8	103.0
Urine 2	–	–	1.58 (± 0.08)	50.0	5.0	1.0	48.67 (± 1.86)	5.27 (± 0.26)	2.56 (± 0.10)	97.3	105.4	98.0
Urine 3	–	–	3.16 (± 0.14)	20.0	2.0	3.0	20.36 (± 0.94)	2.07 (± 0.09)	6.09 (± 0.28)	101.8	103.5	97.7

^a^All human urine samples were collected from the laboratory personnel

^b^Average ± confidence interval, the confidence level is 95%

## Conclusion

In this paper, PVP-GR composite was prepared and exhibited good dispersibility and film-forming properties. A uniform and stable film can be formed on the GCE surface by a simple drop-coating method. The electrochemical behavior of AA, DA and UA on PVP-GR/GCE was explored by cyclic voltammetry. Moreover, with the unique structure of the modified layer and the synergistic effect of GR and PVP, the modified electrode had good catalytic activity for the electrochemical oxidation of AA, DA and UA. Besides, the three peaks could be clearly separated, which can achieve the simultaneous determination of the three small biomolecules. Most importantly, the PVP-GR/GCE was applied to the determination of AA, DA and UA in human urine samples with satisfactory results. The developed methodology was successfully adopted to the simultaneous analysis of these species in human metabolism during early stage of diseases, and also provided a promising strategy in this field. Therefore, it should have a good development prospect.

## Data Availability

All data and materials are fully available without restriction.
